# Nutrients and bioactive compounds as modifiers of neurodegenerative trajectories: molecular mechanisms, translational barriers, and precision nutrition

**DOI:** 10.3389/fnut.2026.1819432

**Published:** 2026-06-03

**Authors:** Gaurav Singh, Gursimran Singh, Shreya, Anjali Kumari, Khadga Raj Aran

**Affiliations:** 1 Department of Pharmacology, ISF College of Pharmacy, Moga, Punjab, India; 2 Department of Pharmacy Practice, ISF College of Pharmacy, Moga, Punjab, India

**Keywords:** bioactive compounds, mitochondrial dysfunction, neurodegenerative disorders, neuroinflammation, nutrient-sensitive neurodegeneration, precision nutrition

## Abstract

The Neurodegenerative diseases (NDs) such as Alzheimer's disease (AD), Parkinson's disease (PD), Multiple sclerosis (MS), and Amyotrophic lateral sclerosis (ALS) are a growing health burden across the world with minimal disease-modifying treatment and therapy. It is emerging that neurodegeneration is not only a progressive loss of neurons, but also a nutrient-sensitive systems-level dysfunction that takes the form of redox imbalance, chronic neuroinflammation, mitochondrial dysfunction, impaired proteostasis, and synaptic loss. The aging brain are more prone to metabolic vulnerability, and subclinical deficiencies in essential nutrients and bioactive dietary compounds may exacerbate cellular stress responses that contribute to disease progression. It summarizes the existing data on the effects of nutrients like vitamins, minerals, polyunsaturated fatty acids, and various phytochemicals in modulating neuronal homeostasis by regulating oxidative signaling, inflammatory cascades, mitochondrial resilience, autophagy, and synaptic plasticity. These nutrient-mediated effects collectively influence neuronal survival, synaptic integrity, and cognitive function by affecting disease susceptibility and progression. Additionally newer metabolites of the marine and microbiome act as new neuroactive agents. The evidence from *in-vitro* and preclinical models, translation to clinical benefit remains inconsistent due to heterogeneity in study design, bioavailability, blood- brain barrier penetration, dosing strategies and disease stage. This review highlights emerging potential of precision nutrition frameworks that integrate nutrigenomics, metabolomics, and microbiome interactions, and individualized metabolic profiling to enable context-dependent and stage-specific interventions. Moreover, conceptualizing neurodegeneration as a nutrient-sensitive, systems level disorder, propose a mechanistically informed and integrative approach that combine targeted nutritional strategies with pharmacological and lifestyle therapies to more effectively modify neurodegenerative trajectories.

## Graphical Abstract

**Figure:**
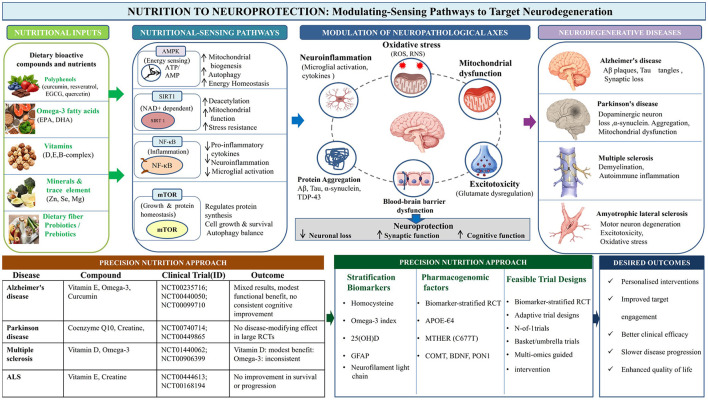

## 1 Introduction

Neurodegenerative diseases (NDs), such as Alzheimer's disease (AD), Parkinson's disease (PD), Multiple sclerosis (MS), Amyotrophic lateral sclerosis (ALS), and others, are increasingly being understood as being complex multifactorial conditions that go beyond single neuron loss (1). The progressive neurodegeneration is the pathological hallmark, growing evidence suggests that such disorders represent a systems-level impairment, which comprises metabolic imbalance, chronic inflammation, mitochondrial dysfunction, disrupted proteostasis, and synaptic failure (2). The prevalence of the conditions in the world keeps increasing due to an aging population, but disease-modifying therapies have not been sufficient and are incapable of preventing the chronic development of diseases. The unresolved gap in therapies highlights the necessity to reconsider prevailing disease models, focusing mostly on downstream protein aggregation and neuronal death. Rather, the convergent mechanistic evidence indicates that neuronal susceptibility develops as a result of accumulated perturbations in cellular networks that mediate redox homeostasis, immune communication, energy generation, and protein quality control (3). It is an energetically demanding part of the body is brain, that requires well-coordinated bioenergetic flux, antioxidant defenses, membrane lipid integrity, enzymatic reactions mediated by micronutrients and dynamic signaling cascades to maintain synaptic transmission and plasticity. Old age people have been linked to low mitochondrial efficiency, dysfunctional glucose metabolism, reduced antioxidant capacity, lipid composition changes and adaptive stress responses. Although there is no overt malnutrition, subclinical deficiencies of vitamins, trace elements, polyunsaturated fatty acids, and bioactive phytochemicals can further increase the oxidative stress, inflammatory priming, and mitochondrial dysfunction that eventually trigger glial inflammatory mediators and impair synaptic integrity. Mitochondrial dysfunction also worsens the production of ATP, enhances proteostatic breakdown, causing the accumulation of misfolded proteins and synaptic loss. These self-organizing processes propose that NDs indicate a progressive breakdown of nutrient sensitive regulatory system as opposed to individual molecular pathologies (4). The necessary nutrients and dietary bio actives have integrative functions, like Vitamin control antioxidant defenses, mitochondrial metabolism, epigenetic methylation and neurotransmitter synthesis (5). The trace elements include iron, zinc, copper, and selenium that are important enzymatic cofactors but cause oxidative damage once regulated. Polyunsaturated fatty acids have an impact on membrane fluidity, lipid-mediated signaling, and inflammatory resolution. The polyphenols, flavonoids, alkaloids, terpenoids, marine and microbiome-derived metabolites alter transcriptional programs, mitochondrial biogenesis, autophagy, and neuroimmune signaling (6). Instead of merely being free radical scavengers, these compounds regulate nutrient-sensitive pathways such as Nuclear factor erythroid 2-related factor 2 (Nrf2), Nuclear factor kappa B (NF-κB), AMP-activated protein kinase (AMPK), Mechanistic target of rapamycin (mTOR), Peroxisome proliferator–activated receptor gamma coactivator 1-alpha (PGC-1α) are determine redox adaptation, inflammatory tone, metabolic flexibility. Throughout experimental studies, it is established that nutritional insufficiency increases synaptic degeneration and mitochondrial dysfunction, and nutrient resilience and cognitive function. The results verify the fact that nutrition is mechanistically integrated into neurodegenerative pathophysiology, although there is strong mechanistic evidence it has been hard to translate into systematic clinical benefit. Human neurodegenerative diseases are characterized by changes over decades and are informed by genetic disparity, metabolic comorbidity, environmental exposures, and lifelong diet (7). Clinical trials frequently use test subjects at late stages of disease, have limited intervention periods, or use single-nutrient supplementation programs that do not mimic the nutrient pathway interactions as seen in preclinical systems. Differences in bioavailability, blood-brain barrier (BBB) penetration, dosing regimens, and underlying nutritional conditions also make interpretation difficult. Furthermore, traditional cognitive and motor measures might be insensitive to sense early metabolic or synaptic alterations. These issues illustrates necessity of biomarker-based stratification, stage-based interventions, and long-term dietary trials, which are less reductionist at the system level. A comparative overview of disease-specific pathological axes, nutrient sensitivity, representative compounds, and clinical evidence is provided in Table 1.

**Table 1:** Disease-specific pathological features, nutrient sensitivity, and clinical evidence in neurodegenerative disorders.

S. No.	Disease	Pathological axis	Nutrient sensitivity	Representative compound	Clinical evidence (trial ID)	Outcome	References
1.	Alzheimer's disease	Amyloid-β, tau pathology, oxidative stress, mitochondrial dysfunction	Antioxidants, anti-inflammatory, methylation support	Vitamin E, omega-3, curcumin	NCT00235716; NCT00440050; NCT00099710	Clinical trials of nutrient-based interventions in Alzheimer's disease have yielded largely inconsistent outcomes. Vitamin E demonstrated modest slowing of functional decline but no significant cognitive improvement. Omega-3 fatty acids failed to show benefit in established disease, likely due to late-stage intervention. Curcumin trials were limited by poor bioavailability and lack of detectable efficacy. These findings suggest that timing, formulation, and patient selection critically influence therapeutic response.	(112–115)
2.	Parkinson's disease	Dopaminergic neuron loss, mitochondrial dysfunction, α-synuclein aggregation	Mitochondrial enhancers, antioxidants	CoQ10, creatine	NCT00740714; NCT00449865	Large randomized trials in Parkinson's disease have failed to demonstrate disease-modifying effects. Coenzyme Q10 did not slow disease progression in the QE3 trial. Creatine supplementation also showed no significant clinical benefit. These findings highlight the translational gap between strong preclinical evidence and clinical outcomes	(138, 139, 141)
3.	Multiple sclerosis	Autoimmune demyelination, chronic neuroinflammation	Immunomodulatory, anti-inflammatory nutrients	Vitamin D, omega-3	NCT01440062; NCT00906399	Nutritional interventions in multiple sclerosis have shown relatively more encouraging outcomes. Vitamin D supplementation demonstrated modest benefits in reducing relapse rates and disease activity. However, omega-3 fatty acids have shown inconsistent or minimal clinical benefit. Variability in baseline vitamin D status and immune activity may influence outcomes	(116, 117)
4.	Amyotrophic lateral sclerosis	Motor neuron degeneration, excitotoxicity, mitochondrial dysfunction	Energy metabolism support, antioxidants	Vitamin E, creatine	NCT00444613; NCT00168194	Clinical trials in amyotrophic lateral sclerosis have shown limited success with nutritional interventions. Creatine did not improve survival or disease progression. Vitamin E also failed to demonstrate significant clinical benefit. These findings may reflect aggressive disease progression and late intervention. Overall, current evidence does not support strong efficacy of nutritional therapies in ALS	(118, 119)

Reconceptualizing neurodegeneration as a nutrient-sensitive disease does not mean that nutritional interventions are sufficient to counteract pathological changes. Instead, it focuses on metabolic resilience as a determinant of neuronal vulnerability that can be modified. A more comprehensive model of disease modification is possible by combining the studies of nutritional science with systems biology, redox signaling, mitochondrial, immunometabolism, and synaptic plasticity. It is a view that promotes a reductionist supplementation change to coordinated, precision based-strategies to match nutrient availability to individual metabolic, genetic and inflammatory profiles. This review highlights new evidence to redefine neurodegenerative disorders as nutrient-sensitive systems-level conditions influenced by metabolic resilience and cellular stress responses. Additionally, combine mechanistic knowledge with the insights on translational perspectives by synthesizing the existing evidence concerning its role as essential nutrients and bioactive compounds in the regulation of redox balance, neuroinflammation, mitochondrial activity, and synaptic integrity and discuss the opportunities and challenges of adopting precision nutrition strategies to manage neurodegenerative trajectories.

## 2 Neurodegeneration as a system-level failure

Neurodegeneration should be conceptualized as the progressive breakdown of a complex system of cells in an environment, instead of the consequence of a single cellular molecular defect, where neurons operate within a metabolically challenging network closely integrating redox homeostasis, inflammatory signaling, energy generation, and protein quality regulation. The main cause of this systems failure is the chronic redox imbalance in which the production of reactive oxygen species (ROS) and nitrogen species predominates over the adaptive ability of the brain to eliminate these species, resulting in the oxidation of lipids, proteins, and nucleic acids. Despite the critical role of physiological redox communication and plasticity in neuronal communication, aging and pathology deactivate adaptive antioxidants responses by disabling glutathione metabolism, antioxidant enzyme activity and redox sensitive transcriptional programs, diminishing the capacity of the brain to respond to metabolic challenges (8). Microglia and astrocytes that normally play metabolic support synaptic cleaning and immune surveillance roles gradually change to maladaptive phenotypes with long-term oxidative stress, releasing excess pro-inflammatory cytokines, chemokines, and reactive species that increase neuronal injury (9). This inflammatory conditions worsens with time leading to glial dysfunction affecting neurotransmitter recycling, neurotropic support and Blood-Brain Barrier (BBB) integrity enabling peripheral immune mediators to further up-regulate central inflammation and supporting a pathological loop. Notably, the redox balance and inflammation are closely controlled by cell metabolism and nutrient- sensitive signaling pathways which suggest that metabolic inadequacy may cause the system to shift to chronic inflammation due to mitochondrial activity as the stressors progressively mount lead to a loss to their bioenergetic based upon which neuronal survival and synaptic activity depend.

**Figure 1:**
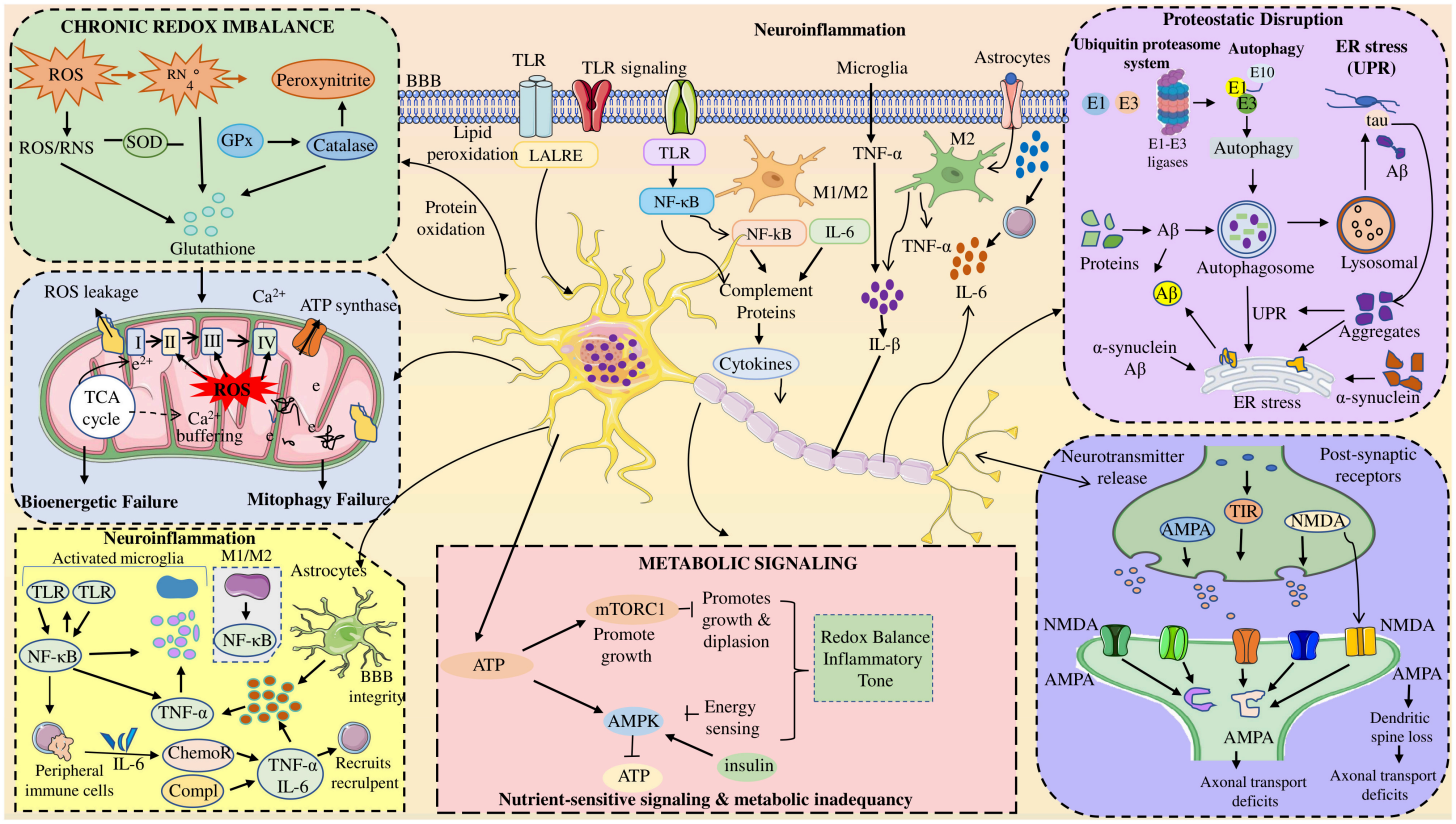
This figure illustrates a systems-level view of neurodegeneration, showing how multiple interconnected processes drive neuronal damage. Chronic redox imbalance leads to excessive production of reactive oxygen and nitrogen species, overwhelming antioxidant defenses and causing oxidative damage to lipids, proteins, and mitochondria. This results in mitochondrial dysfunction, reduced ATP production, and increased oxidative stress, further aggravating cellular injury. Simultaneously, activation of neuroinflammatory pathways through microglia and astrocytes—mediated by the NF-κB pathway—leads to the release of pro-inflammatory cytokines such as TNF-α and IL-6, disrupting blood–brain barrier integrity. In parallel, proteostatic mechanisms including the ubiquitin–proteasome system and autophagy become impaired, resulting in the accumulation of toxic protein aggregates such as amyloid-β, tau, and α-synuclein. Dysregulation of metabolic signaling pathways, particularly the AMPK pathway and mTOR, further contributes to energy imbalance and inflammatory signaling. These combined disturbances ultimately lead to synaptic dysfunction, impaired neurotransmission, and neuronal loss, forming a self-perpetuating cycle that underlies the progression of neurodegenerative diseases.

Mitochondrial dysfunction constitutes a threshold phenomenon wherein the upstream redox and inflammatory imbalances are converted into systemic cell death as neurons rely virtually entirely on oxidative phosphorylation to satisfy an extraordinary amount of energy requirements in the body. Mitochondria in neurodegenerative conditions have a lower efficiency of electron transport, defective ATP production, altered calcium buffering and leakage of reactive species, which diminishes axonal transport, synaptic vesicle cycling, and electrical excitability (10). This protein homeostasis is directly affected by this energetic insufficiency, in which both the ubiquitin-proteasome system and autophagy-lysosomal pathways depend on sufficient ATP and proper signaling by nutrient-sensitive signals, including mTOR and AMPK (11). This breakdown of the proteostatic systems leads to the accumulation of misfolded and aggregation-prone proteins as toxic species that interfere with intracellular trafficking, mitochondrial dynamics and synaptic architecture. Protein aggregates are frequently secondary effects of metabolic and redox stress over the long-term and increase the pathogenesis of cell malfunction by physically and functionally stressing neurons that have already been impaired by other causes (12). The cumulative impact of all these network failures is most likely to be found at the synapse, where adaptability and connectivity require high energetic demands, turnover of all proteins, and redox regulation. The loss of synaptic structures is thus among the first systems-level collapses, and synaptic loss is more closely associated with cognitive and motor loss than neuronal death itself (13). With the degeneration of synaptic networks, the process of communication between circuits fails; functional degradation gradually occurs, manifesting itself in neurodegenerative disorders. All together, these processes indicate that neurodegeneration is a result of the failure of an interdependent metabolic and signaling network where redox imbalance, neuroinflammation, mitochondrial failure, and proteostatic disruption are similarly tied together and thus the idea that metabolic and nutritional resilience can alter disease pathways and not just focus on downstream pathological phenotypes. Figure 1 illustrates the Systems-level network driving neurodegeneration through interconnected metabolic and signaling failure.

## 3 Essential nutrients as modulators of neural homeostasis

### 3.1 Vitamins as regulators of redox balance, epigenetics, and neurotransmission

The basic neuro-regulators vitamins play an important role in maintaining redox balance, controlling epigenetic pathways, and facilitating neurotransmitter synthesis in highly metabolically active neural tissue. Since the brain requires an excessive amount of systemic oxygen and has lipid-rich membranes prone to peroxidation, preservation of antioxidant defense mechanisms is essential for neuronal survival (14). Vitamin C and E, along with other antioxidant vitamins, act synergistically to counter reactive oxygen species and reduce lipid peroxidation, further maintaining membrane integrity, mitochondrial stability, and synaptic activity. It also involves redox recycling of vitamin C, and increasing vitamin E and glutathione-dependent antioxidant systems production, and enhancing intracellular oxidative stress responses (15). Simultaneously, vitamin B-complex indirectly maintains redox-homeostasis by facilitating mitochondrial oxidative phosphorylation and Nicotinamide adenine dinucleotide/Flavin adenine dinucleotide (NAD^+^/FAD)-dependent mitochondrial enzymatic processes that are necessary to neuronal bioenergetics (16). Besides acting as antioxidants, vitamins are crucial in epigenetic regulation systems that dictate the neural gene expression over the lifetime. The carbon metabolism centrally involves folate and vitamin B12, which produce S-adenosylmethionine used in Deoxyribonucleic acid (DNA) and histone methylation interactions, neuronal differentiation, myelin survival and synaptic plasticity. These mechanisms of methylation are more interfered with by homocysteine, a neurotoxic metabolite that is associated with cortical degeneration and cognitive impairment (17). Although vitamin A and D also increase epigenetic action by transcriptional regulations that are mediated by nuclear receptors, which regulate genes implicated in neurotropic signaling, calcium homeostasis, immune regulation and synaptic remodeling (18). Vitamins are also required cofactors in neurotransmitters, as well synaptic control, particularly, pyridoxal phosphate (vitamin B6), which is necessary in making dopamine, γ-aminobutyric acid, and histamine, which directly affects neural circuits excitation inhibition homeostasis (19). Vitamin C is also implicated in synthesis of catecholamine and mechanism of release of neurotransmitters. Thiamine and niacin stimulate the production of acetylcholine and NAD^+^ dependent signaling synaptic plasticity and cognition pathways (15). All these interrelated biochemical positions designate the vitamins as integrative regulators of neural homeostasis, the oxidative defense mediators, the chromatin regulators, the chromatin regulators and mediators of neural transmission in well-orchestrated neurobiological pathways.

#### 3.1.1 Fat-soluble vitamins: signaling and membrane integrity

Lipid-soluble micronutrients, which are absorbed in the stomach and are carried to the central nervous system and other organs through lipoproteins, are the fat-soluble vitamins (A, D, E, and K). Their lipophilicity enables them to be efficiently built into neuronal membranes and access intracellular and nuclear receptors, connecting them transcriptional regulation and intracellular signaling to membrane structure (20). The retinol and all-trans retinoic acid (RA) ensure gene expression through retinoic acid receptors (RARs) and retinoid X receptors (RXRs) facilitated by vitamin A. These ligand receptor controlled transcription factors establish on the retinoic acid response element of the promoter regions of genes that participate in neuronal differentiation, synaptic plasticity and dopaminergic signaling. The adult brain RA signaling regulates striatal dopamine receptor expression as well as hippocampal synaptic remodeling, which is of high importance to neurodegenerative disorders. Disruption of this retinoid signaling has been associated with the subsequent disruption in synaptic homeostasis, amplified amyloidogenic processing, and disturbed in synaptic homeostasis, amplified amyloidogenic processing, and disrupted neuroinflammatory signaling (21, 22). Vitamin D receptor (VDR) is a promoter that has been shown to be expressed in neurons, astrocytes, and microglia, and heterodimerizes with the RXR to mediate the transcription of calcium homeostasis, antioxidant defense, and neurotropic support genes (140). Calcitriol suppresses Nf-κB activation stabilizes intracellular calcium response, and stimulates nerve growth factor (NGF) and glial cell line-derived neurotropic factor (GDNF) production to prevent excitotoxic and inflammatory injury (23). This keeps the membranes fluid, and conforms polyunsaturated fatty acids, which is a key factor in synaptic vesicle fusion, and provides the integrity of mitochondrial membranes (24). Additionally its antioxidant action, vitamin E influences the signaling pathways involving kinase including MAPK and protein kinase C, which are dependent on kinase and requires the presence of this vitamin, and regulates expression of apoptosis and inflammatory genes (25). Similarly, vitamin K works in neural membrane by being involved in sphingolipid metabolism and gamma-carboxylation of proteins including growth arrest-specific protein 6 (Gas6). Gas6-TAM receptor signaling is active and induces survival signaling through PI3K/Akt and alleviates microglial reactivity that contributes to neuronal viability and membrane stability (26). Collectively, fat-soluble vitamins regulate receptor-mediated transcriptional pathways and maintain membrane density and place them as neuronal modulators of neurodegenerative disease resilience.

#### 3.1.2 Water-soluble vitamins: metabolic and methylation control

Vitamin-B and C and complex are water-soluble vitamins whose central functions are in neuronal metabolic flux, one-carbon transfer reactions, redox buffering, and neurotransmitter biosynthesis, so they have multilayered control of neural homeostasis. Thiamine (B1), riboflavin (B2), and niacin (B3) are obligatory cofactors of mitochondrial dehydrogenases and electron transport chain proteins through thiamine pyrophosphate, flavin adenine dinucleotide, and nicotinamide adenine dinucleotide (NAD^+^), respectively, supporting oxidative phosphorylation and ATP synthesis in metabolically active neuronal pathways (27). It is associated with reduced levels of NAD^+^ and the operation of flavoproteins, thereby damaging sirtuin signaling, mitochondrial biogenesis, and DNA repair, which links bioenergetic failure in old age with vitamin B complex deficiency (28). These vitamins regulate intracellular homocysteine levels, which increase excitotoxicity, oxidative stress, and endothelial dysfunction by regulating the pathways of methionine synthase and cystathionine 2-synthase (29, 30). Vitamin B-complex concentrations maintain the patterns of DNA and histone methylation that are essential to neuronal gene expression, synaptic plasticity, and myelin maintenance. Disruption of these methylation-dependent processes has been linked with hippocampal atrophy, white matter degeneration, and impaired cognitive functioning (27, 31). Vitamin C (ascorbate) play important role in metabolic control mediated by B-vitamins with potent antioxidant activity and enzyme cofactor activity. It replenishes depleted glutathione, keeps iron and copper in catalytically active monooxygenase conformations, and promotes the production of dopamine 2-hydroxylase-dependent catecholamines (32, 33). Ascorbate also regulates epigenetic processes by acting as a cofactor in ten-eleven translocation (TET) dioxygenases in DNA demethylation (34). Together, B-complex vitamins and vitamin C combine mitochondrial energetics and methylation-dependent gene regulation and redox stability to mediate the metabolic framework that supports neuronal survival and cognitive health.

### 3.2 Minerals and trace elements: neuroprotection vs. neurotoxicity

Minerals and trace elements are involved in highly regulated molecular pathways that support neuronal excitability, redox homeostasis, mitochondrial respiration, and synaptic transmission, but the small physiological concentrations over which they act make the central nervous system highly sensitive to both deficiency and toxicity (35). Iron exemplifies this duality as it is an essential cofactor for cytochromes and iron-sulfur cluster-containing enzymes within the electron transport chain, and it supports oxidative phosphorylation and neurotransmitter synthesis. However, redox-active ferrous iron catalyzes Fenton chemistry that generates hydroxyl radicals, leading to initiation of lipid peroxidation, impairing membrane integrity, and precipitating ferroptotic neuronal death (36). Such dysregulations which are commonly marked by the abnormal expression of transferrin receptors and ferritin sequestration of nutrients have been witnessed in substantia nigra neurons and they are mechanistically coupled to agglomerate the α-synuclein aggregations and mitochondrial impairments, hence linking the iron mal-regulation to proteotoxic stress (37). Simultaneously, zinc is involved in both structural stabilization and dynamical signaling. In the physiological state, it helps in antioxidant defense by stabilizing superoxide dismutase (SOD) and controlling thiol buffering by metallothionein (38). However, an overload of zinc in synapses in the process of excitotoxicity leads to destabilization of the mitochondrial membrane and activation of NADPH oxidate dependent ROS, and enhances apoptotic cascades, and the further conversion of modulator ion into an injury mediator (39). Copper also serves as another example of balance between the metabolic need and redox liability. Participation as cofactor in the cytochrome c oxidase and dopamine 2-hydroxylase, copper maintains mitochondrial respiration and catecholaminergic neurotransmission, but copper perturbations trafficking proteins such as ATP7A and ATP7B disrupt intracellular disruption and initiate aberrant redox cycling, which increases oxidative stress effects on amyloidogenic processing (40, 41). Besides redox-active metals selenium has a neuroprotective property since it is incorporated into selenoproteins, including glutathione peroxidases and thioredoxin reductases, that sustain thiol homeostasis and neutralize the impact of hydrogen peroxide (42). Deficiency of selenium increases the neuroinflammatory signaling and inhibited synaptic plasticity, but adequate amounts of selenoproteins maintain mitochondrial stability (43). Collectively, the integration of transporters, storage proteins, and redox-sensitive pathways determines whether these essential elements function as neuroprotective cofactors or transition into catalysts of oxidative injury and inflammatory amplification within neurodegenerative contexts. Table 2 shows the essential nutrients homeostasis through specific molecular targets, including transcriptional regulators, mitochondrial pathways, redox signaling systems, and synaptic plasticity mechanisms. Their effects are context-dependent with both deficiency and toxicity potentially contributing to neuronal dysfunction and neurodegenerative processes.

**Table 2:** Essential nutrients, neural targets, and context-dependent effects.

S. No	Nutrient	Molecular targets/pathways	Physiological role in neural homeostasis	Dysregulation/excess mechanism	Neurodegenerative implication	References
1.	Vitamin A (retinoic acid)	RAR/RXR nuclear receptors; CREB signaling; synaptic plasticity genes	Regulates neuronal differentiation, synaptic remodeling, hippocampal plasticity	Impaired retinoid signaling disrupts gene transcription and synaptic maintenance	Cognitive decline, impaired neurogenesis	(20–22)
2.	Vitamin D	VDR–RXR complex; calcium channels; Nrf2 signaling; neurotrophins	Modulates Ca^2+^ homeostasis, anti-inflammatory signaling, and antioxidant gene expression	Deficiency enhances neuroinflammation and oxidative stress	Increased risk of AD, PD	(18, 23, 140)
3.	Vitamin E (α-tocopherol)	Lipid membranes; PKC modulation; lipid peroxidation chains	Terminates lipid peroxidation; preserves membrane fluidity	Deficiency promotes membrane lipid oxidation and ferroptosis	Accelerated neuronal degeneration	(24, 25)
4.	Vitamin K	Sphingolipid metabolism; Gas6 signaling	Maintains membrane integrity and myelin stability	Low levels impair sphingolipid synthesis	White matter vulnerability	(20, 26)
5.	Vitamin B6 (Pyridoxal-5-phosphate)	Amino acid decarboxylases; neurotransmitter synthesis enzymes	GABA, dopamine, and serotonin synthesis	Deficiency alters excitatory–inhibitory balance	Seizure susceptibility, cognitive dysfunction	(19, 27, 29)
6.	Vitamin B9/B12	One-carbon metabolism; SAM-dependent methylation; DNA methyltransferases	Epigenetic regulation, homocysteine clearance	Hyperhomocysteinemia induces oxidative and vascular injury	Cognitive decline, brain atrophy	(17, 30, 31)
7.	Vitamin C (ascorbate)	ROS scavenging; dopamine β-hydroxylase cofactor; glutamate uptake	Maintains redox balance, modulates neurotransmission	Depletion increases oxidative neuronal injury	Synaptic dysfunction	(15, 33, 34)
8.	Iron	ETC cytochromes; Fe-S cluster enzymes; ferritin; transferrin receptor	Oxidative phosphorylation, neurotransmitter synthesis	Fenton reaction → hydroxyl radicals → ferroptosis; altered ferritin storage	α-synuclein aggregation, mitochondrial dysfunction (PD)	(36, 37)
9.	Zinc	Zinc-finger transcription factors; NMDA receptor modulation; metallothionein	Synaptic plasticity; antioxidant enzyme stabilization	Excess synaptic Zn^2+^ → mitochondrial depolarization; NADPH oxidase activation	Excitotoxic neuronal death	(35, 38, 39)
10.	Copper	Cytochrome c oxidase; dopamine β-hydroxylase; ATP7A/ATP7B	Mitochondrial respiration; catecholamine synthesis	Aberrant redox cycling; oxidative stress; amyloid modulation	Tau phosphorylation; amyloidogenesis	(40, 41)
11.	Selenium	Glutathione peroxidases; thioredoxin reductases	Thiol redox homeostasis; mitochondrial protection	Deficiency increases neuroinflammation and ROS	Synaptic impairment	(42, 43)

## 4 Bioactive compounds beyond antioxidants

### 4.1 Polyphenols and flavonoids as modulators of inflammation and autophagy

Natural products like flavonoids and polyphenols serve as antioxidants, neutralizing ROS and reducing oxidative stress. Polyphenols like resveratrol and curcumin have been demonstrated to reduce oxidative stress in neurons. These natural compounds increase the activity of antioxidant enzymes like catalase and superoxide dismutase (SOD), which protect neurons from oxidative damage and maintain cognitive function. Polyphenols are widely found in fruits, vegetables, wine, and tea that effectively reduce oxidative stress and neuroinflammation, two of the primary causes of dementia, because of their strong anti-inflammatory and antioxidant properties (44). Resveratrol that is present in grapes and red wine alleviates oxidative stress and regulates inflammation to help preserve the wellbeing of neurons. *In vitro* research indicates that resveratrol (5–25 μM) can alleviate the generation of ROS in neurons in rat hippocampus exposed to nitric oxide free radical donors (45). As per study demonstrated, resveratrol also maintains cognitive ability because it reduces microglial activity to reduce neuroinflammation in animals with AD (46). Resveratrol effectively suppressed LPS induced proinflammatory factor (NO, TNF-α, and IL-1β) generation in neuron glia cultures, providing dopamine neurons in Parkinson, disease with a high level of neuroprotection. The production of these proinflammatory factors produced by LPS in the supernatant of neuron glia cells also significantly reduced by the administration of resveratrol (47). Curcumin, which is derived from turmeric (*Curcuma longa* L., Fam: Zingiberaceae), has been well studied for its neuroprotective properties. It reduces tau tangles, inhibits Aβ aggregation, and enhances cognitive performance in AD models (48). Another polyphenol, epigallocatechin gallate (EGCG) from green tea (*Camellia sinensis* (L.) Kuntze, Fam: Theaceae), protects neurons by reducing inflammation and scavenging free radicals. Furthermore, flavonoids like kaempferol and quercetin inhibit the activation of microglia and astrocytes, which reduces the production of pro-inflammatory cytokines (49). These actions reduce brain inflammation and may slow the onset of neurodegenerative diseases. In multiple sclerosis, quercetin promotes the proliferation and development of oligodendrocyte progenitor cells (OPCs) by preventing apoptosis and protecting them via the PI3K/Akt pathway. It lowers the microgliosis and astrocytes through the inhibition of inflammatory mediator's production that inhibits the astrocytes and microglial (50). Curcumin is an effective anti-oxidant neuroinflammation through the inhibition Nf-κB, which is a requirement of inflammatory reaction in the brain (51). Ginsenosides from *Panax ginseng* C. A. Mey also reduce inflammation in PD with the mediators of pro-inflammatory cytokines and labial microglia (52).

### 4.2 Alkaloids and terpenoids targeting neuronal signaling

Alkaloids are nitrogenous secondary metabolites that are produced by both plants and microbes and have at least one nitrogen atom in a ring. They are well known for their ability to modify neurotransmitter systems and enhance cognitive performance (53). Yang et al. (54) suggest that Trevis, Fam: Lycopodiaceae, contains an acetylcholine-deploying chemical, Huperzine A, which can enhance memory and cognitive functions and acetylcholine levels in AD. Berberine is an anti-inflammatory and glucose-metabolizing alkaloid obtained from the *Berberis* species and could be used in AD and PD models. Berberine prevents Aβ-induced microglial activation through the suppressor of cytokine signaling 1 (SOCS1), further demonstrating the value of berberine in the pathophysiology of AD (55, 56). Moreover, berberine reduces endoplasmic reticulum stress, which consequently decreases cognitive impairments because endoplasmic reticulum stress is required to transmit through the hyperactivation of GSK-3β and phosphorylation of eukaryotic translation initiation factor-2α (eIF2α) using activation of PRKR-like endoplasmic reticulum kinase (PERK) and may even curb neurological issues by limiting this metabolic pathway (57).

Their antioxidant and anti-inflammatory qualities support the preservation of neural integrity. Ginkgolides, which are produced by *Ginkgo biloba* L. (Fam: *Ginkgoaceae*), have antioxidant properties and enhance blood flow, preventing conditions like dementia and cognitive decline (58). *G. Biloba* inhibits the synthesis of inflammatory chemicals by preventing the release of proinflammatory mediators and cytokines, such as nitric oxide, prostaglandin E2 (PGE2), TNF-α, interleukin 6 (IL-6), and IL-1β. Additionally, it promotes the suppression of nuclear factor kappa-light-chain-enhancer of activated B cells (NF-κB), cyclooxygenase-2 (COX-2), and 5-lipoxygenase (5-LOX), further prevents oxidative stress by blocking peroxiredoxin-1; moreover, it suppresses the expression of adhesion factors, such as vascular cell adhesion molecule 1 (VCAM-1) and intercellular adhesion molecule 1 (ICAM-1), which are essential for leukocyte adhesion to the blood vessel surface and subsequent migration to inflammation. In this sense, it actively works to prevent neuroinflammation (59). Limonene, one of nature's most prevalent terpenes, is present in neroli, cumin, bergamot, caraway oil, dill oil, and citrus peel oil. This phytochemical can induce apoptosis by various biochemical effects, mostly anticancer, including negatively regulating antiapoptotic proteins and positively regulating pro-apoptotic factors. Its anti-inflammatory, anti-cancer, and antioxidant properties provide neuroprotection against a range of neurological disorders, including AD, stroke, and cerebral ischemia (60–62). In particular, limonene inhibits rotenone (ROT), which inhibits complex-1 of the mitochondrial electron transport chain. This can reduce locomotor impairments associated with PD and create a condition that resembles the behavioral and neuropathological signs of the illness. It also stops aberrant protein deposition, stops mitochondrial dysfunction, and initiates abnormal cell death (63).

### 4.3 Marine-derived and microbial metabolites as emerging neuroactive agents

There are several special natural materials found in marine habitats that may be used to treat NDs like AD and PD. Marine organisms, such as algae and sponges, produce phlorotannins and omega-3 fatty acids because they have evolved to harsh environmental conditions. Among the most widely investigated classes are marine peptides and protein hydrolysates, which suppress NF-κB and MAPK signaling (p38, ERK, JNK), thereby reducing transcription of pro-inflammatory mediators such as IL-1β, IL-6, TNF-α, iNOS, and COX-2 (64). In parallel, their antioxidant properties counteract mitochondrial dysfunction and apoptotic cascades through ROS scavenging and metal chelation. Bioactive peptides from *Euphausia superba* (Antarctic krill) and *Stichopus japonicus* (sea cucumber) have been shown to improve cognitive impairment in experimental models through the regulation of acetylcholine metabolism, long-term potentiation, and inhibition of oxidative damage (65). The carotenoid astaxanthin, which has both ketone and hydroxyl groups, is found in microalgae, shellfish, shrimp, trout, and salmon. It is well recognized for being a strong antioxidant with neuroprotective qualities that help in preventing oxidative stress and inflammation by triggering the antioxidant network, which comprises catalase and superoxide dismutase (SOD). Moreover, astaxanthin protects neurons in numerous neurodegenerative diseases, including AD and PD, by inhibiting inflammatory mechanisms, making it a potential anti-inflammatory drug in the CNS (66, 67). Phenol-tannins isolated in brown algae are effective antioxidants, which can potentially counteract oxidative stress and neuroinflammation by inhibiting TNF-α, IL-1β, NADPH oxidase, as well as by activating Sirt-3 and PGC-1α pathways (68). Bryostatin, a protein kinase C (PKC) potentiator derived from the brown bryozoan *Bugula neritina*, enhances synaptogenesis and cognitive and behavioral impairments in rodent models of AD (69). Fucoidan is a sulfated polysaccharide derived from the brown algae *Undaria pinnatifida* that has demonstrated to decrease the effects of Aβ_1_-_42_ and hydrogen peroxide-induced cytotoxicity in PC12 cells and points to its antioxidant and anti-amyloid properties (70).

**Figure 2:**
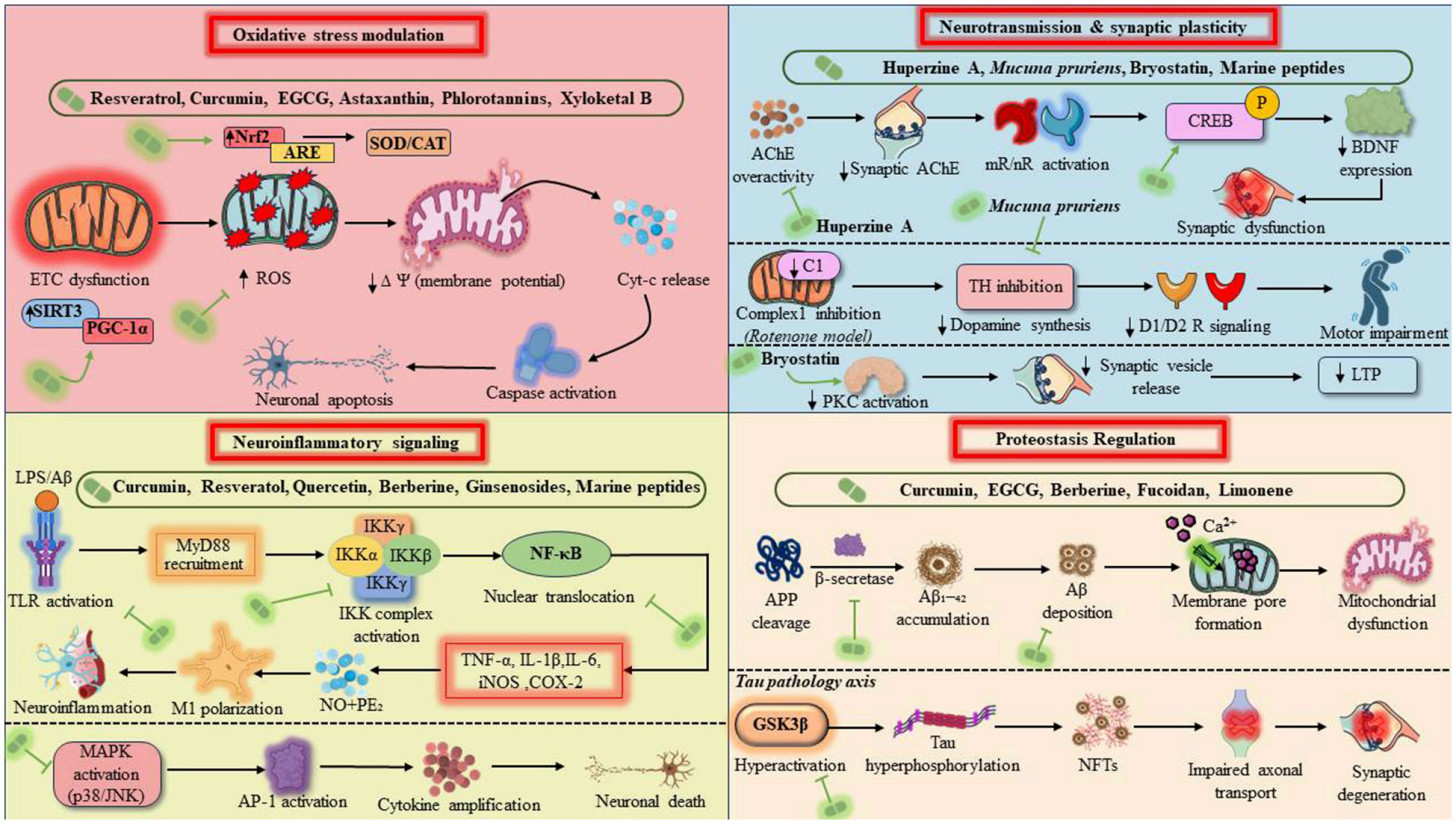
This figure illustrates how dietary bioactive compounds exert multi-target neuroprotective effects across four key pathological domains of neurodegeneration. The oxidative stress module, compounds such as resveratrol, curcumin, EGCG, and astaxanthin activate the Nrf2 pathway and antioxidant enzymes (SOD, CAT), improving mitochondrial function and reducing ROS-mediated apoptosis. In neurotransmission and synaptic plasticity, agents like huperzine A and *Mucuna pruriens* enhance cholinergic and dopaminergic signaling, increase CREB-mediated BDNF expression, and improve synaptic function, thereby counteracting motor and cognitive deficits. The neuroinflammatory signaling panel shows inhibition of TLR/MyD88-mediated activation of the NF-κB pathway, leading to reduced production of pro-inflammatory cytokines (TNF-α, IL-1β, IL-6) and suppression of microglial activation. Finally, in proteostasis regulation, compounds such as curcumin and EGCG inhibit amyloid-β accumulation, modulate β-secretase activity, and reduce tau hyperphosphorylation via GSK3β, thereby preventing synaptic degeneration and mitochondrial dysfunction. Overall, the figure highlights that nutritional bioactives act through integrated molecular pathways to simultaneously reduce oxidative stress, inflammation, protein aggregation, and synaptic dysfunction in neurodegenerative diseases.

Xyloketal B, isolated from the mangrove fungus along the South China Sea coast, possesses anti-inflammatory, anti-apoptotic, and antioxidant properties by scavenging free oxygen radicals and reducing damage to neurons and endothelial cells. Zhao et al. (71) demonstrated the neuroprotective effects of xyloketal B in 2009 using an *in vitro* model of PC12 cells that had been subjected to glucose and oxygen deprivation. This study showed that mitochondrial ROS generation was markedly elevated after oxygen and glucose deprivation, whereas xyloketal B significantly reduced the MitoSOX signal intensity, suggesting that mitochondria may represent a primary target of its anti-apoptotic action in neuronal cells (71). These marine natural products demonstrated multiple biological activities supporting their therapeutic potential and providing a foundation for future research. Figure 2 illustrates the Multimodal neuroprotective mechanisms of bioactive natural compounds in neurodegenerative diseases.

## 5 Nutrient driven signaling pathways in neurodegenerative disorders

### 5.1 Nutrient regulation of oxidative stress and redox signaling

**Figure 3:**
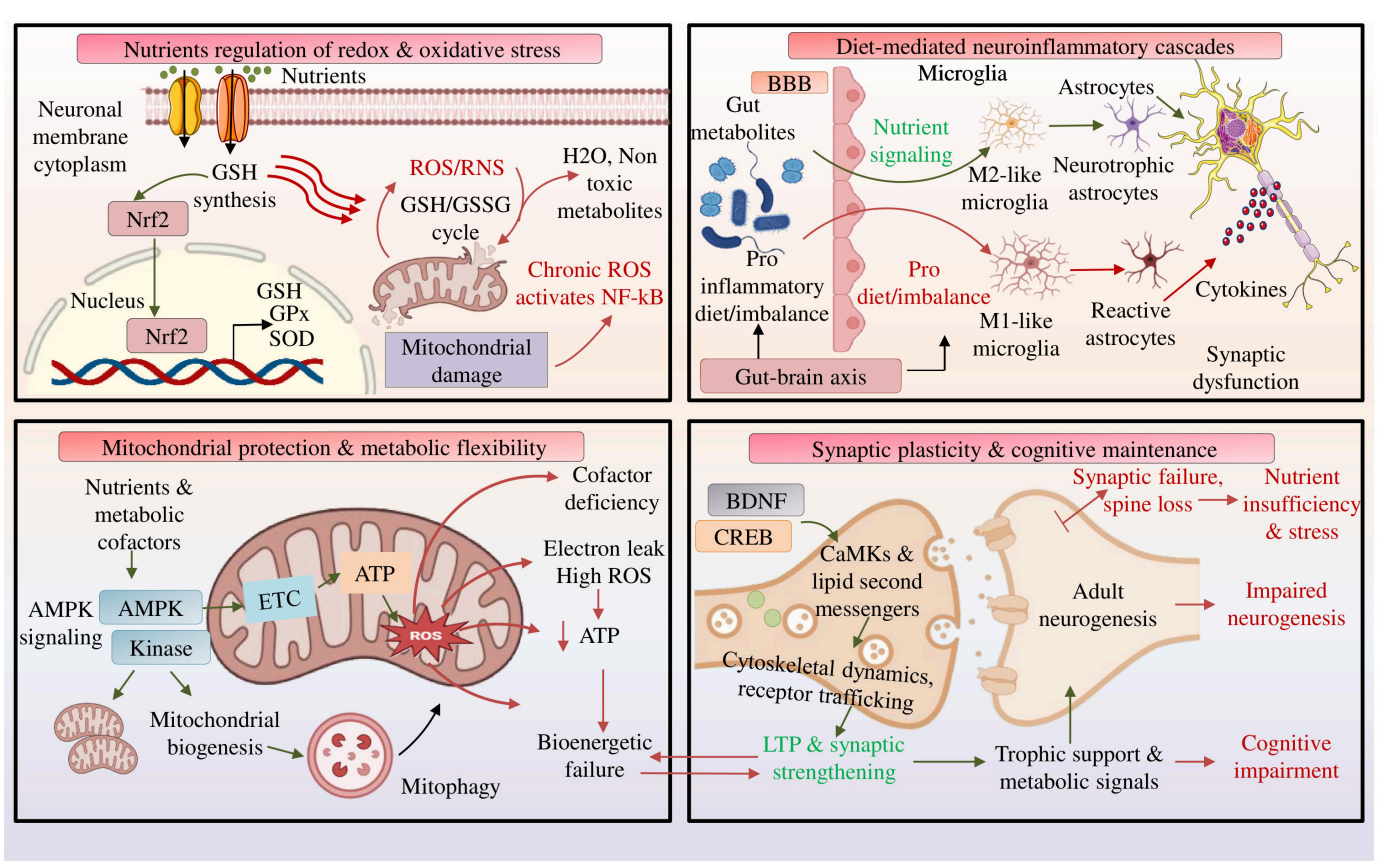
Nutrient-driven regulation of interconnected pathological pathways in neurodegenerative disorders. Nutrient availability and dietary patterns modulate four interdependent pathological axes that collectively determine neuronal resilience in neurodegenerative diseases. (1) Redox regulation and oxidative stress control: Nutrients activate Nrf2-dependent antioxidant defenses, enhance GSH synthesis, and support SOD and GPx activity to neutralize excess ROS/RNS and limit mitochondrial damage. (2) Diet-mediated neuroinflammatory cascades: Pro-inflammatory dietary imbalance and gut-derived metabolites activate NF-κB signaling, promote M1-like microglial polarization and reactive astrocytosis, elevate cytokine release, and compromise BBB integrity via the gut-brain axis. (3) Mitochondrial protection and metabolic flexibility: Nutrient-sensitive AMPK signaling preserves ETC efficiency, supports mitochondrial biogenesis and mitophagy, reduces electron leak-induced ROS, and maintains ATP-dependent neuronal bioenergetics. (4) Synaptic plasticity and cognitive maintenance: Nutrient-responsive pathways involving BDNF, CREB, CaMKs, and lipid-derived second messengers sustain LTP, cytoskeletal dynamics, receptor trafficking, and adult neurogenesis, whereas nutrient insufficiency accelerates dendritic spine loss, synaptic failure, and cognitive impairment. Collectively, the figure highlights diet and nutrient signaling as upstream modulators of redox balance, inflammatory tone, mitochondrial quality control, and synaptic integrity in neurodegenerative disorders. AMPK, AMP-activated protein kinase; BBB, blood-brain barrier; BDNF, brain-derived neurotrophic factor; CaMKs, calcium/calmodulin-dependent protein kinases; CREB, cAMP response element-binding protein; ETC, electron transport chain; GSH, reduced glutathione; GPx, glutathione peroxidase; LTP, long-term potentiation; NF-κB, nuclear factor kappa-B; Nrf2, nuclear factor erythroid 2-related factor 2; ROS/RNS, reactive oxygen/nitrogen species; SOD, superoxide dismutase.

Oxidative stress is a pathogenic driver in the most major NDs such as AD, PD, ALS, and HD, where endogenous redox buffering mechanisms are overwhelmed by excessive levels of reactive oxygen and nitrogen species and the impairment of neuronal integrity occurs. Numerous postmortem and *in vivo* experiments repeatedly show that there is an early loss of glutathione and a decrease in antioxidant enzymes activity in susceptible brain areas, including the hippocampus in Alzheimer's disease and the substantia nigra in Parkinson's disease, suggesting that redox imbalance is a cause of neuron loss rather than the end-stage pathology (72). Experimental models also indicate that dietary deficiency of redox supportive nutrients enhances oxidative damage, increases the rate of mitochondrial damage and enhances neuronal inflammatory signaling, and that nutrient restored dietary deficiencies improve redox resilience and neuronal survival (140). The trace elements that are crucial cofactors to the glutathione peroxidases and superoxide dismutase respectively e.g., selenium and zinc are often abnormal in neurodegenerative disorders, and both deficiency and mislocalization are thought to contribute to the dysfunction of the redox enzymes (73) as shown in Figure 3. The clinical and preclinical evidence demonstrates that the antioxidant defense can be partially restored and oxidative damage attenuated by correcting such imbalances, which underlines the idea that neuronal redox homeostasis requires an active support of enzymes based on sufficient nutrients as opposed to the passive antioxidant scavenging taking place.

**Figure 4:**
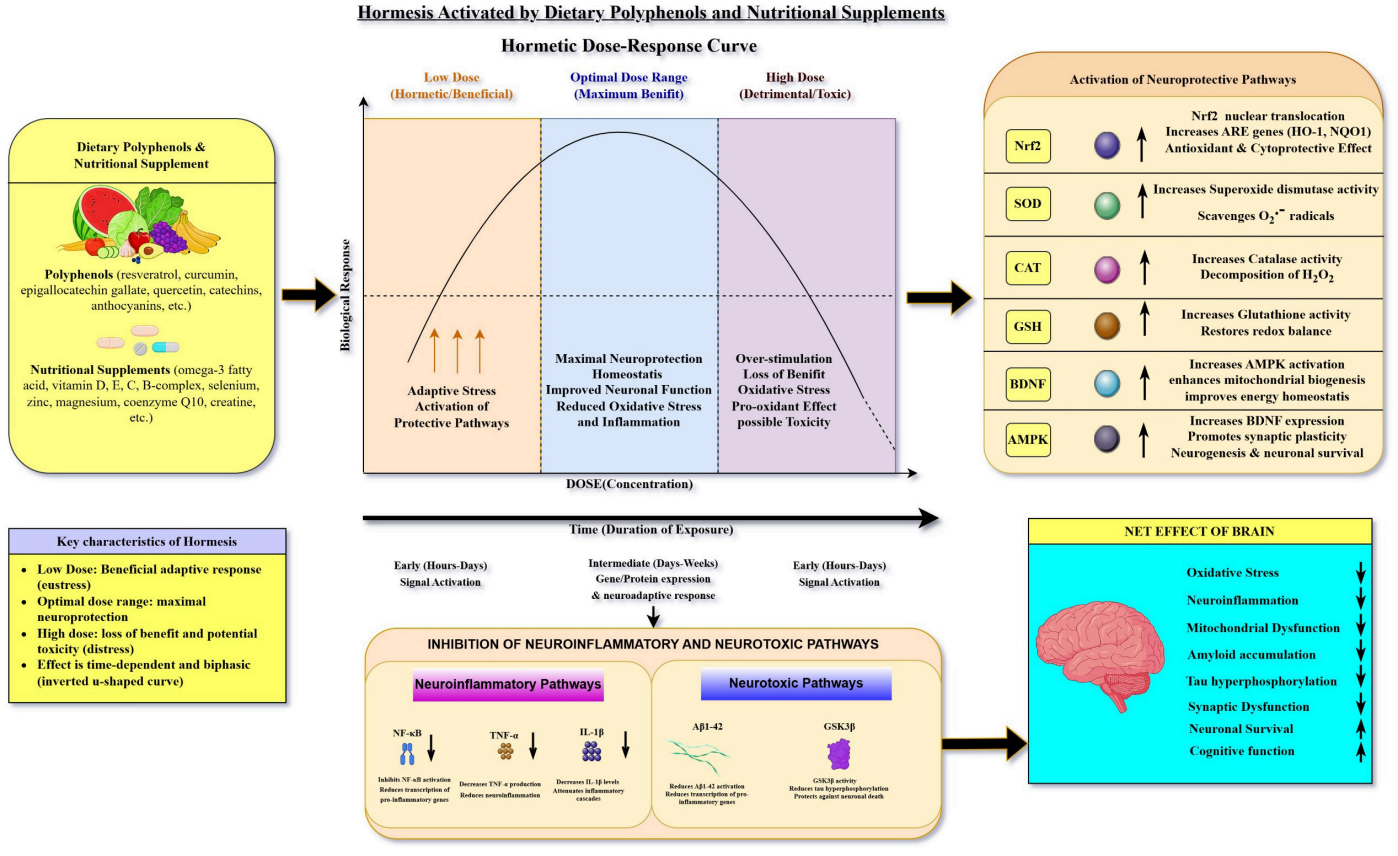
This figure illustrates the concept of hormesis in neuroprotection, showing how dietary polyphenols and nutritional supplements exert dose-dependent effects on brain health. At low doses, compounds such as resveratrol, curcumin, and omega-3 fatty acids induce mild cellular stress that activates protective pathways, including the Nrf2 pathway, antioxidant enzymes (SOD, CAT, GSH), and metabolic regulators like AMPK pathway and BDNF, leading to enhanced mitochondrial function, redox balance, and neuronal survival. At an optimal dose range, these effects are maximized, resulting in reduced oxidative stress, decreased neuroinflammation, and improved synaptic and cognitive function. However, at high doses, the benefits decline and may become detrimental, causing oxidative stress and toxicity due to overstimulation. The figure also highlights that these effects are time-dependent, involving early signaling events followed by gene and protein expression changes. Overall, hormetic activation suppresses neuroinflammatory pathways (e.g., NF-κB, TNF-α, IL-1β) and neurotoxic processes (e.g., Aβ1–42, GSK3β), ultimately promoting neuroprotection and slowing neurodegenerative progression.

In addition to direct antioxidant effects, nutrients have an extensive influence on redox signal networks that mediate gene expression, mitochondrial activity, and inflammatory responses in an overall systems-wide disease pathology (74). The Nrf2 signaling pathway has become one of the most nutrient-sensitive pathways of cellular redox regulation, with control of transcription of antioxidant, detoxification, and mitochondrial maintenance genes. There is a neurodegenerative disease model, evidence that malfunctioning Nrf2 activation is associated with an increase in oxidative load, neuroinflammatory progression, and cognitive and motor outcomes, and dietary bioactive compounds to activate Nrf2 signaling lead to an increase in endogenous antioxidant systems and neuronal resistance (75). Research involving toxin-induced and genetic models of Parkinson's and Alzheimer's disease show that nutrient-enhanced redox regulation enhances mitochondrial signaling, maintains synaptic behavior and neurodegenerative phenotypes. Redox signaling has also found to interact with the inflammatory pathways with the oxidative stress stimulating transcription factors like NF-κB that trigger chronic glial stimulation and cytokine release, redox restorations through nutrient have been demonstrated to reduce this loop of inflammatory amplification (76). Beyond the direct antioxidant effects, the growing evidence suggests that these bioactive compounds exert their effects through hormetic modulation of cellular stress responses. These natural bioactive compounds, including flavonoids, polyphenols, and marine-derived metabolites, modulate redox homeostasis primarily through activation of the Nrf2 pathway and downstream phase II antioxidant enzymes such as SOD, catalase, and glutathione systems. These compounds suppress pro-inflammatory mediators, including IL-1β, IL-6, TNF-α, iNOS, and COX-2 which further reduces neuroinflammatory signaling. Their biological effects follow a hormetic dose-response relationship, where low-to-moderate doses induce adaptive cellular stress responses that enhance antioxidant effect and neuronal resilience, whereas excessive doses may impair redox balance and promote toxicity (77, 140). This biphasic behavior highlights that bioactive compounds function as hormetic nutrients capable of modulating mitochondrial function, reducing neurotoxicity, and enhancing stress resilience pathways in a dose-dependent manner. Additionally, emerging evidence indicates that the gut-brain axis contributes to these effects, as dietary bioactives and their microbiota-derived metabolites regulate systemic redox balance and neuroinflammatory signaling. The growing evidence indicates that sustained, moderate exposure optimally activates Nrf2-driven cytoprotective mechanism and improves cognitive outcomes, whereas supraphysiological dosing may disrupt endogenous antioxidant systems (78). These findings highlights dose optimization as a key determinant in translating bioactive compounds into effective neuroprotective strategies. Figure 4 illustrates that hermetic dose-responses of dietary polyphenols showing optimal-dose activation of neuroprotective pathways and suppression of neuroinflammatory and neurotoxic mechanisms. Together results suggest oxidative stress in NDs indicates the failure of nutrient-regulated redox signaling and not an inevitable oxidative injury, and nutrition is central to predisposition of the neurons. Instead of random antioxidant supplementation approach growing support in the evidence base that suggests adaptive redox signaling, metabolic flexibility, and mitochondrial resilience as a reasonable way of altering the progression of neurodegenerative disease.

### 5.2 Diet-mediated control of neuroinflammatory cascades

A characteristic of neurodegenerative disorders is chronic neuroinflammation, which is now being recognized as a diet-sensitive, metabolically controlled process as opposed to a secondary response to neuronal injury. Cross-model preclinical studies of the AD, PD and other related disorders all indicate a significant effect of dietary composition on the microglial and astrocytic activation state, thus determining whether the process of inflammatory signaling is resolved adaptively or leads to persistent neurotoxicity (79). Poor or pro-inflammatory diets worsen oxidative stress and trigger innate immune response such as NF-kB and inflammasome activation leading to prolonged release of pro-inflammatory cytokines, reactive nitrogen species and lipid mediators that destabilize synaptic integrity and neuronal survival. Conversely, nutrient-enriched diet intervention suppress glial priming, maintain synaptic structure and delay the progression of the disease in experimental models (80). Dietary cues in glial cells trigger immunometabolic changes, mediating transition between oxidative and glycolytic states, which are the direct determinants of inflammatory phenotype. Exposure to nutrient imbalance selectively induces a glycolysis-dependent and pro-inflammatory phenotype in microglia that is marked by defective phagocytic clearance of protein aggregates and overproduction of cytokines whereas balanced nutrient signaling promotes anti-inflammatory and neurotrophic function as shown in Figure 3. Similarly, astrocytes react to nutritional signals in changing lipid metabolism, antioxidant activity, and recycling of neurotransmitters and regulate neuronal excitability and inflammation tone (81). Clinically, the correlation of diet- mediated regulation of neuroinflammation with neurodegenerative consequences is supportive but inconsistent, which is due to the complexity of the human dietary exposure, diversity of the disease and prolonged disease latency (82). Dietary approaches to reduce oxidative stress, amyloid-β aggregation, and neuroinflammation have been effective in animal models of AD (83). Various phytochemicals, such as polyphenols, omega-3 fatty acids and antioxidant vitamins, have been reported to regulate important signaling pathways, such as the Nrf2 pathway and NF-κB pathway, thereby reducing oxidative stress and inflammation (84). The study shows that clinical result vitamin E supplementation has shown some evidence of slowing functional decline in clinical trials, but not significant cognitive improvement (85). Similarly, omega-3 fatty acids have failed to improve outcomes in advanced Alzheimer's patients, possibly due to starting treatment too late. Resveratrol, while able to activate SIRT1 and AMPK pathways in animal models, has not significantly improved symptoms, possibly due to low bioavailability and high metabolism rates (86). These observations highlight the importance of disease progression, pharmacokinetics and variability in patient responses. Most randomized controlled studies on inflammation using single-nutrient supplementation have found only small or inconclusive results, indicating the weakness of reductionist approaches that do not consider synergies between nutrients in the diet and nutrient status as well as the permeability of the blood-brain barrier. There is rising evidence of emerging translation support that whole-dietary and long-term dietary pattern intervention are better moderators of neuroinflammatory cascades, in part by the regulation of the brain-axis (87). The diet-induced changes in the composition of gut microbiota and the production of microbial metabolites affect peripheral immune response, BBB function and central glial reaction which offer another inflammatory control. Combined, these results support the idea that diet-triggered neuroinflammation control can be achieved by coordinated metabolic, immune and microbial interactions, which means that long-term context-specific dietary-based interventions have a higher potential to alter neurodegenerative dysfunction than temporary anti-inflammatory treatment.

### 5.3 Mitochondrial protection, metabolic flexibility and neuronal survival

Mitochondrial dysfunction is a fundamental pathogenic pathway in neurodegenerative diseases, which combines upstream damages of oxidative stress, chronic inflammation and nutrient imbalance into a condition of bioenergetics failure that reduces neuronal viability (88). The neurons have a unique reliance on efficient oxidative phosphorylation in the mitochondria to support synaptic transmission, axonal transport and ionic homeostasis and thus make them highly susceptible to mitochondrial signal disruption. The preclinical models of AD, PD, Huntington's disease (HD), ALS and electron transport chain all show early disruption of electron transport chain activity, diminished ATP production, appropriate mitochondrial dynamics, as well as excessive generation of reactive species before neurons are lost (89). AMPK-regulated nutrient-dependent signaling pathways play leading roles in controlling mitochondrial biogenesis, antioxidant defense and mitophagy, hence ensuring good mitochondrial quality control (90). It is experimentally shown that perturbation of these pathways enhances neurodegenerative phenotypes, and dietary regulation of mitochondrial signaling maintains synaptic plasticity and retards cell dysfunction. Metabolic cofactor, fatty acid, and micronutrient deficiency reduce the efficiency of the mitochondria, encourage leakage of electrons, and increase redox stress establishment of a feed-forward loop where energetic deficiency further worsens adaptive stress responses (91) as shown in Figure 3. These findings place mitochondria not only as passive targets of neurodegeneration, but also as active integrators of nutrient-driven signaling that determines neuronal survival. On a translational scale, growing evidence indicates that nutritional regulation of mitochondrial signaling can provide a plausible approach to the improvement of neuronal survival, however, clinical validation is yet to be confirmed (92). The diet-based interventions that increase metabolic flexibility in preclinical systems have been shown to improve mitochondrial respiration, stabilize the membrane potential and promote the selective elimination of dysfunctional mitochondria by a process known as mitophagy, limiting the buildup of dysfunctional organelles (93). These consequences can be characterized as the enhanced functioning of the synapsis, a decrease in the neuroinflammation and a longer delay in the disease development, which highlights the interrelation of mitochondrial dysfunction with isolated metabolic supplements that have yielded diverse results with many failings to provide solid disease-modifying effects despite excellent mechanistic support. These differences can probably be seen as the complexity of human neurodegenerative disease, in which dysfunction of mitochondria is caused by the combination of aging, genetic predisposition, chronic inflammation, and lifelong dietary habits and not the deficiency of a specific biochemical pathway (94). Accurate clinical and observational evidence is quickly mounting that indicates the significance of maintaining dietary habits that encourage metabolic robustness, as opposed to temporary treatments that focus on enhancing mitochondrial production. Besides, mitochondrial signaling is overlapped with the metabolic health of the whole body, such as insulin sensitivity and lipid metabolism, which connects the peripheral metabolic failure with vulnerability of brain neurons (95). From this approach, mitochondrial protection in neurodegeneration is best understood as a network-level result of nutrient-driven signaling that maintains energy flexibility in the face of chronic stress. All these perspectives support the idea that the renewal of metabolic flexibility and mitochondria quality regulation via specific nutritional treatments can be one of the key strategies to maintain neuronal survival and delay neurodegenerative disease progression, especially when they are combined with other interventions aimed at redox balance and neuroinflammatory load.

### 5.4 Synaptic plasticity, neurogenesis and cognitive maintenance

Synaptic plasticity is the cellular mechanism of learning memory, and cognitive adaptability and its impairment is among the first events in neurodegenerative disease with the greatest functional relevance (96). Synapses are highly metabolically active structures in the brain that demand constant energy, maintain redox, remodel dynamic membranes, and protein turnover is tightly regulated in order to maintain activity-directed plasticity (97). Signaling pathways that are driven by nutrients play a role in sustaining such processes because nutrients control neurotransmitter production, synaptic vesicle cycling, receptor expressing, and cytoskeletal dynamics via metabolic and signaling intermediates. Preclinical studies in AD and PD models consistently show that delivery insufficiency exacerbates dendritic spine loss, impairs long-term potentiation, and accelerates synaptic failure, whereas nutrient-rich or metabolically supportive dietary interventions preserve synaptic architecture and improve cognitive abilities (98). Nutrients have the mechanistic effect of modulating important plasticity-related signaling pathways, such as brain-derived neurotropic factor (BDNF), Cyclic AMP response element–binding protein (CREB), calcium-dependent kinases, and lipid-derived second messengers, which combine metabolic signals and neuronal activity to promote synaptic strengthening and remodeling. Interruption of these nutrient-sensitive pathways impairs the ability of synaptic flexibility, reduces the oxidative stress, inflammatory mediator and mitochondrial dysfunction threshold to damage neuronal communication, strengthening a feed-forward loop of synaptic susceptibility and cognitive impairment as shown in Figure 3. Adult neurogenesis, especially in the hippocampal dentate gyrus, helps in cognitive flexibility, memory formation, and network adaptability and it is very sensitive to metabolic and nutritional status (99). Neurogenic capacity is impaired in NDs and aging due to disruption of neuronal integration by impaired neural progenitor proliferation, altered differentiation programs, and inflammatory microenvironment (100). Critically, neurogenesis is functionally related to synaptic plasticity, since newly formed neurons are more plastic and take an over-proportional part in circuit remodeling during learning, therefore enhancing the cognitive effects of nutrient-driven signaling (101). Evidence of nutrition-mediated improvement of synaptic plasticity and neurogenesis as contributing to cognitive maintenance is still suggestive in clinical settings, as it is hard to assess synaptic and neurogenic outcomes in humans or on time scales as long as structural remodeling needs to be (102). Observational studies implicate nutrient-rich dietary habits with maintained cognitive functioning and lower risk of dementia, but randomized experimental studies of specific nutrient supplementation have produced inconsistent results, probably because of variability in disease progression, initial nutritional status, and multifacetedness of synaptic signaling pathways. The PD nutritional approaches have targeted mitochondrial dysfunction, oxidative damage and inflammation. Neuroprotective effects of coenzyme Q10, creatine, and omega-3 fatty acids have been observed in experimental models, through improvements in mitochondrial bioenergetics and suppression of inflammatory agents (103). However, these mechanisms have yet to translate to clinical benefit and randomized studies with coenzyme Q10 and creatine have not shown disease-modifying activity (104). Similarly, omega-3 fatty acids have shown variable results in clinical trials reflect the fact that these studies were conducted at a time when significant loss of dopaminergic neurons has already occurred, and that nutritional status has not been considered in the design of the studies (105). These findings suggest the importance of early intervention and individual patient characteristics for successful nutritional interventions. Taken collectively, there is an indication of breakdown in nutrient-sensitive plasticity and neurogenic signaling and not necessarily irreversible loss of synapses. Through the maintenance of synaptic plasticity, endorsing circuit renewal, and consolidating cognitive reserve, nutrient-mediated plasticity and neurogenesis processes become essential element of long term cognitive reserve in an aggregate system also comprising of redox balance, inflammatory regulation, and mitochondrial health. Table 3 shows the Molecular targets and cellular specificity of nutrients and bioactives.

**Table 3:** Molecular targets and cellular specificity of nutrients and bioactive compounds in neurodegenerative disorders.

S. No.	Nutrient	Bioactive compound	Primary molecular targets	Key signaling pathways modulated	Predominant responsive cell types	Functional outcomes relevant to neurodegeneration	References
1	Selenium, cysteine, vitamins C and E	Selenoproteins, glutathione (GSH), ascorbate, α-tocopherol	Glutathione synthesis, redox enzymes	Nrf2–ARE, redox-sensitive kinases	Neurons, astrocytes	Enhances endogenous antioxidant buffering and limits lipid, protein, and DNA oxidation; supports synaptic integrity and delays oxidative stress driven neuronal dysfunction	(120)
2	Fruits, vegetables, plant-derived foods	Polyphenol, flavonoids	Keap1, NF-κB, MAPKs	Nrf2 activation, inflammatory suppression	Microglia, neurons	Suppresses chronic microglial activation and inflammatory cytokine release while promoting adaptive stress-response signaling that preserves neuronal viability	(121)
3	Omega-3 fatty acids (DHA, EPA)	Docosahexaenoic acid (DHA), Eicosapentaenoic acid (EPA)	Membrane phospholipids, lipid mediators	PPAR signaling, lipid-derived cascades	Neurons, astrocytes, microglia	Improves membrane fluidity and synaptic plasticity, attenuates neuroinflammatory lipid signaling, and supports cognitive and motor function maintenance	(122)
4	B-complex vitamins (B1, B6, B9, B12)	Thiamine pyrophosphate (TPP), Pyridoxal-5-phosphate (PLP), folate derivatives, methylcobalamin	Methylation enzymes, mitochondrial complexes	One-carbon metabolism, energy signaling	Neurons, oligodendrocytes	Maintains mitochondrial energy production and neurotransmitter synthesis; reduces homocysteine-mediated neurotoxicity and supports myelin integrity	(27)
5	Vitamin D	Calcitriol (1,25-dihydroxyvitamin D3)	Vitamin D receptor (VDR)	Neuroimmune and Ca^2+^ signaling pathways	Microglia, astrocytes, neurons	Modulates neuroimmune responses and calcium homeostasis, reducing inflammatory-mediated neuronal injury and supporting synaptic stability	(123)
6	Iron, copper, zinc	Cu^2+^, Fe^2+^/Fe^3+^, Zn^2+^ ions	Metal-dependent enzymes, mitochondrial proteins	Redox and bioenergetic signaling	Neurons, microglia	Essential for enzymatic and mitochondrial function, but dysregulation promotes oxidative stress, protein aggregation, and ferroptotic neuronal death	(124)
7	Amino acids (glutamate, glycine, taurine)	Glutamate, glycine, taurine	Neurotransmitter receptors, antioxidant synthesis	Excitatory–inhibitory balance, redox control	Neurons, astrocytes	Regulates synaptic transmission and excitability; imbalance contributes to excitotoxicity and redox dysregulation in neurodegenerative states	(125)
8	Dietary fiber, prebiotics, probiotics	Short-chain fatty acids (SCFAs), tryptophan metabolites	Immune receptors, endothelial tight junction proteins	Gut-brain axis signaling	Microglia, endothelial cells	Modulates peripheral-to-central immune signaling, maintains BBB integrity, and attenuates chronic neuroinflammation linked to disease progression	(126)
9	Glucose, ketone bodies	β-Hydroxybutyrate (BHB), acetoacetate	SIRT1, AMPK, mitochondrial respiratory complexes, insulin receptor signaling	AMPK–SIRT1–PGC-1α axis, insulin signaling pathway, mitochondrial biogenesis pathways	Neurons, astrocytes	Enhances mitochondrial efficiency, improves synaptic plasticity, reduces insulin resistance–mediated neuronal dysfunction, decreases oxidative stress, and supports neurotransmitter balance	(127)

## 6 From bench to beside: why translation often fails

Preclinical models have been crucial in the discovery of diet-influenced processes of relevance to neurodegenerative conditions, and offer regulated systems where the impact of food ingredients on oxidative stress, neuroinflammation, mitochondrial activity and synaptic plasticity can be assessed in a controlled manner. *In vitro* and animal systems provide the possibility of an accurate manipulation of nutrient supply and signaling pathways, enabling the establishment of causal relationships that are not possible in human studies (106). Such models have produced highly interesting mechanistic arguments on the importance of nutrition in the context of regulating neurodegenerative pathways; nevertheless, their translational application is limited by their nature (107). Human NDs develop over decades and are a result of the interplay of aging, genetic susceptibility and metabolic impairment and environmental conditions, where experimental systems usually depend on acute injury, loss of single gene or accelerating disease models (2). These methods can either overestimate the extent of nutritional effects or overlook structure and lifespan species differences complicate extrapolation, and even experimental models prove to be essential in generating hypothesis and identification of pathways, might overrate effectiveness and predictability of nutritional interventions in case are used in heterogeneous groups of patients. Most of the studies enroll participants at late stages of the disease, when the circuit disruption and neuronal loss could be less susceptible to metabolic manipulation, and thus would be less likely to show an observable benefit (92). Another key issue is dosing strategies, with nutrient bioavailability, BBB penetration, and tissue-specific metabolism frequently being ill-defined and mononutrient supplementation may be incapable of interacting with the network of pathways that are observed in preclinical studies. Besides, overdosing or non-physiological dosing may interfere with adaptive redox and metabolism signaling that would compromise possible advantages (108). To address these shortcomings ever more attention has been given to nutraceuticals, functional foods and real-world dietary interventions that are more consistent with habitual dietary exposure and provide multi-pathway action (109). Although such methods are promising and correspond to the systems-level character of neurodegeneration, they cause difficulties in the fields of standardization, compliance, and confounding lifestyle factors. The bench-to-bedside gap will thus be bridged with integrative clinical approaches that will integrate mechanistic biomarkers based on preclinical considerations. It can be crucial to consider nutrition as a long-term tool that can be change metabolic resilience and not a short-term therapeutic one when interpreting experimental results into meaningful clinical results in managing neurodegenerative diseases. Table 4 states about the Clinical evidence, outcomes, and key translational limitations.

**Table 4:** Clinical evidence and translational limitations of nutritional interventions in neurodegenerative disorders.

S. No.	Nutrient	Intervention type	Target neurodegenerative disorders	Clinical evidence (study type)	Reported outcomes	Key translational limitations	References
1	Vitamins C, E	Single-nutrient supplementation	AD, PD	Randomized control trial or observational study	Modest reduction in oxidative stress and lipid peroxidation biomarkers; limited or no consistent improvement in cognitive scores or disease progression, with effects varying by dose and disease stage	Non-specific antioxidant dosing, limited BBB penetration, disruption of physiological redox signaling	(128)
2	Vitamins B6, B9 (folate), B12	Targeted vitamin supplementation	AD, MCI	RCTs, cohort studies	Significant reduction in plasma homocysteine levels; cognitive slowing observed primarily in individuals with baseline deficiency or elevated homocysteine	Benefits largely restricted to deficient populations; late-stage intervention limits impact	(129)
3	DHA, EPA (omega-3 fatty acids)	Lipid supplementation	AD, PD	RCTs, epidemiological studies	Mild improvements in memory, executive function, and synaptic biomarkers in early-stage disease; anti-inflammatory effects observed in peripheral markers	Disease-stage dependence; variability in dose, formulation, and bioavailability	(130)
4	Vitamin D	Hormone-like micronutrient supplementation	AD, PD	Observational studies, small RCTs	Associations with reduced cognitive decline, improved neuroimmune profiles, and lower inflammatory markers; inconsistent cognitive benefit across trials	Confounding lifestyle factors; lack of large, long-duration randomized trials	(131)
5	Polyphenols (e.g., flavonoids, resveratrol)	Nutraceutical/bioactive supplementation	AD, PD	Pilot clinical trials, dietary intervention studies	Improved antioxidant capacity and reduced inflammatory biomarkers; modest or no consistent effects on cognitive endpoints	Poor standardization, low oral bioavailability, short intervention duration	(132)
6	Ketone bodies/medium-chain triglycerides	Metabolic dietary intervention	AD, epilepsy-associated neurodegeneration	Clinical trials, metabolic studies	Improved cerebral glucose metabolism, enhanced ketone utilization, and short-term improvements in cognitive performance	Adherence challenges; long-term safety and sustainability concerns	(133)
7	Mixed nutrients (whole-diet patterns)	Mediterranean/MIND diet	AD, dementia	Longitudinal cohort studies	Lower incidence of cognitive decline and dementia; preservation of executive function and memory over long follow-up periods	Observational design limits causal inference; lifestyle and socioeconomic confounders	(134)
8	Prebiotics, probiotics	Microbiome-targeted intervention	AD, PD	Early-phase clinical studies	Modulation of gut microbiota composition, reduced systemic inflammation, and changes in gut–brain signaling markers	High inter-individual microbiome variability; limited mechanistic and CNS-specific endpoints	(135)
9	Multinutrient formulations	Combined nutrient intervention	AD, MCI	Randomized controlled trials	Modest stabilization of cognitive performance and functional measures in early disease; limited efficacy in advanced stages	Complex formulations obscure mechanism attribution; heterogeneous trial designs	(137)

### 6.1 Polyphenol-based nanoformulations for improving bioavailability and clinical translation

One of the major limitations underlying the failure of dietary polyphenols to translate from preclinical efficacy to clinical efficacy is their poor bioavailability, rapid metabolism, and limited ability to cross the BBB. The novel nanoformulation strategies have been developed to enhance the pharmacokinetic and pharmacodynamic profiles of polyphenols. These include nanoliposomes and phytosomes, solid lipid nanoparticles (SLNs), and nanostructured lipid carriers (NLCs), which improve solubility, stability, and targeted delivery of bioactive compounds. Nanoencapsulation protects polyphenols from premature degradation and facilitates their absorption which resulting to increase systemic bioavailability and enabling higher concentrations of unmetabolized active compounds to reach the brain. The preclinical *in vivo* studies demonstrated that these delivery systems increase brain uptake, improve mitochondrial function, and reduce neuroinflammatory and oxidative stress markers more effectively than free compounds. Moreover, growing evidence suggests that nano-formulated polyphenols may act synergistically with pharmacological agents, further enhancing therapeutic efficacy in clinical settings (110, 111). These innovative approaches represent an important step toward overcoming translational barriers and converting dietary polyphenols into clinically viable neuroprotective interventions.

## 7 Precision nutrition in neurodegenerative disease management

Precision nutrition is an exciting approach to tackling the variability seen in neurodegenerative diseases, but its use is currently theoretical without the support of biomarkers, genetic stratification and practical trial designs. Precision nutrition requires consideration of individual variability in biochemical and genetic factors, as well as disease progression, to move beyond a “one-size-fits-all” approach.

One crucial element is the identification of patients who will respond to specific nutritional interventions through stratification biomarkers. Plasma homocysteine concentrations, for instance, can indicate dysfunction of the one-carbon metabolism and direct supplementation with B vitamins. The omega-3 index serves as a biomarker for long-chain fatty acid levels and may assist in determining who may benefit from omega-3 fatty acid supplementation. Likewise, plasma 25-hydroxyvitamin D [25(OH)D] is essential for identifying vitamin D deficiency and can guide immunomodulatory treatments such as in Multiple sclerosis. New biomarkers, such as fecal short-chain fatty acids (SCFAs) reflect gut bacteria and its impact on the gut-brain axis, while markers for neurodegeneration such as glial fibrillary acidic protein (GFAP) and neurofilament light chain (NfL) reflect injury and disease progression. Beyond biochemical markers, pharmacogenomic factors are important in predicting responses to nutritional therapies. For example, individuals carrying the APOE-ε4 allele, a key genetic risk factor for Alzheimer's disease, may have altered cholesterol metabolism, impacting responses to omega-3 fatty acid supplements. Polymorphisms in MTHFR gene may alter folate metabolism and homocysteine levels and therefore influence the response to methyl-donor nutrients. Likewise, variants in fatty acid desaturase genes (FADS1/2) may affect *de novo* fatty acid synthesis and response to dietary lipid supplements. By considering such genetic information in the design of studies, this allows a more personalized therapeutic approach.

To bring precision nutrition to the clinic, new trial designs are needed. Randomized controlled trials, in which a single intervention is applied to a broad population, may not identify effects in sub-groups. Rather, stratified trial designs should be used, in which participants are screened for specific biomarker or genetic characteristics. For instance, an omega-3 fatty acid supplementation trial in Parkinson's disease may be improved by enrolling individuals who have low omega-3 index and high inflammatory markers, thus enhancing the chance of observing a beneficial effect. Likewise, vitamin D trials in multiple sclerosis could target patients with low baseline vitamin D levels and assess immune outcomes. Adaptive trial designs, with flexible treatment strategies, may also be applied to improve the success of such trials.

Together, these strategies transform precision nutrition from a theoretical possibility to a clinical reality. Through biomarker-based stratification, genetic profiling and tailored trial designs, future research can enhance the translational potential of nutritional interventions and support personalized approaches to treatment in neurodegenerative disorders.

## 8 Limitations of the current review

Despite providing a comprehensive overview of nutrient-driven signaling pathways and their relevance in NDs, various limitations should be acknowledged. This review is inherently subject to selection bias, given the absence of a systematic methodology of literature inclusion, which may lead to preferential emphasis on specific pathways or compounds. Moreover, a considerable proportion of the evidence discussed is derived from preclinical *in vitro* and *in vivo* studies, which may not directly translate to human clinical settings due to differences in physiology, bioavailability, and dosing considerations. The variability in study designs, populations, and outcome measures across the existing literature further limits the ability to extract definitive conclusions regarding efficacy. Additionally, growing areas such as the gut-brain axis and hermetic dose-response relationships remain insufficiently validated in large-scale clinical trials. The lack of standardized dosing strategies and heterogeneity in nutrient formulations, particularly for bioactive compounds such as polyphenols, also limits reproducibility and clinical applicability. Addressing these limitations will require well-designed clinical trials, standardized interventions, and integrative approaches to facilitate the translation of these findings into effective therapeutic strategies.

## 9 Conclusion and future directions

NDs such as AD, PD, MS, and ALS are increasingly recognized as multifactorial disorders defined not only by progressive neuronal loss but also by systemic metabolic dysregulation. The collective mechanistic and translational evidence synthesized in this review supports a paradigm shift in which neurodegeneration is conceptualized as a nutrient-sensitive, systems-level failure involving redox imbalance, chronic neuroinflammation, mitochondrial dysfunction, impaired proteostasis, and synaptic loss. In this context, bioactive compounds and nutrients such as essential vitamins, trace elements, polyunsaturated fatty acids, polyphenols, terpenoids, and marine- and microbiome-derived metabolites cannot be considered only passive antioxidants or supplementary compounds. Instead, they act as dynamic regulators of transcriptional programs, mitochondrial quality control, autophagic flux, inflammatory tone, and synaptic plasticity. Their actions target nutrient-sensitive signaling pathways that coordinate stress adaptation, metabolic flexibility, and neuroprotection. Collectively, these understandings lead to the redefinition of nutrition as a mechanistically constitutive part of neurodegenerative disease biology as opposed to a secondary supportive approach.

Despite robust preclinical evidence, consistent clinical benefits have yet to be achieved, but standardized reporting frameworks, standardized dosing strategies, and mechanistically aligned outcome measures are essential. The stage-specific intervention designs, including those targeting prodromal or early metabolic dysfunction, have the potential to be more disease-modifying in comparison to the trials performed at a late stage. The biomarker-guided stratification incorporating metabolic, inflammatory, and redox markers should be prioritized to identify responsive subgroups. The combination of nutrigenomics, metabolomics, lipidomics, and microbiome profiling with detailed clinical phenotyping represents an important approach. These multi-omic approaches can clarify inter-individual variability, define nutrient-gene, and enable precision-based dietary interventions based on biological context. The longitudinal, multimodal clinical trials combining dietary strategies with pharmacological therapies, physical activity, and lifestyle modulation will be necessary to evaluate synergistic effects on disease progression. Simultaneously, improvements in formulation science, such as nanocarrier systems and targeted delivery platforms, could enhance bioavailability and central nervous system accessibility of bioactive compounds, bridging mechanistic potential and clinical relevance. It is also crucial to develop intermediate biomarkers that indicate mitochondrial functioning, redox equilibrium, synaptic integrity, and neuroinflammation, to identify a therapeutic response earlier than it is too late to detect structural deterioration. Advancing a precision nutrition based on system biology, translational rigor, and individualized stratification offers a promising framework to improve metabolic resilience and potentially alter neurodegenerative mechanisms. Instead of being adjugative-supportive, nutrition can emerge as a determining factor of neuronal vulnerability and recovery and be at the crossroads of metabolism, inflammation, and circuit integrity.
